# An Overview of GIS-Based Modeling and Assessment of Mining-Induced Hazards: Soil, Water, and Forest

**DOI:** 10.3390/ijerph14121463

**Published:** 2017-11-27

**Authors:** Jangwon Suh, Sung-Min Kim, Huiuk Yi, Yosoon Choi

**Affiliations:** 1 Energy Resources Institute, Pukyong National University, Busan 48513, Korea; jangwonsuh@hanmail.net; 2 Research Institute of Energy Resources, Seoul National University, Seoul 08826, Korea; snuhyrule@hanmail.net; 3 Korea Institute of Geoscience and Mineral Resources, Daejeon 34132, Korea; yihuiuk@kigam.re.kr; 4 Department of Energy Resources Engineering, Pukyong National University, Busan 48513, Korea

**Keywords:** mine hazards, geographic information systems (GIS), soil contamination, water pollution, deforestation, abandoned mine

## Abstract

In this study, current geographic information system (GIS)-based methods and their application for the modeling and assessment of mining-induced hazards were reviewed. Various types of mining-induced hazard, including soil contamination, soil erosion, water pollution, and deforestation were considered in the discussion of the strength and role of GIS as a viable problem-solving tool in relation to mining-induced hazards. The various types of mining-induced hazard were classified into two or three subtopics according to the steps involved in the reclamation procedure, or elements of the hazard of interest. Because GIS is appropriated for the handling of geospatial data in relation to mining-induced hazards, the application and feasibility of exploiting GIS-based modeling and assessment of mining-induced hazards within the mining industry could be expanded further.

## 1. Introduction

**Figure 1:**
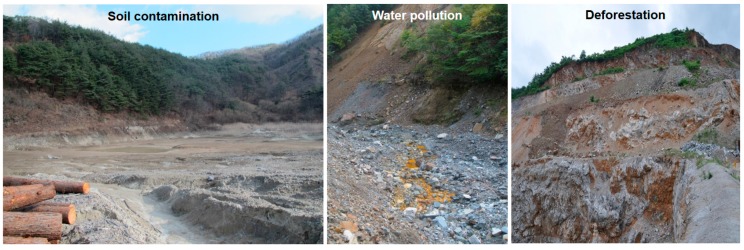
Photographs of representative mining-induced hazards associated with soil (Samgwang mine, Korea), water (Gagok mine, Korea), and forest (Ssangyong limestone mine, Korea). All three photographs were taken by the authors.

Mining within a sustainable framework is important for the welfare of human beings. However, the mining industry has often been perceived negatively because it can be hazardous to both public health and safety, and cause damage to the surrounding environment, including the land, soil, water, and forests at local, regional, and global levels [1]. Mining-induced hazards encompass any of the dangers peculiar to the prospecting for and extraction of coal and minerals. Such hazards include, but are not limited to, land subsidence, soil contamination by heavy metal pollutants or mine tailings, water pollution, inundation by water, deforestation, slope failure, spontaneous combustion, explosion of released gas, inhalation of dust and poisonous gases, and abandoned facilities (Figure 1).

To mitigate or eliminate the potential negative environmental impacts and risks associated with mining activities, an examination of both the extent and the level (degree) of hazards based on continuous assessment within a spatial context is necessary [2]. For this work, the application of geographic information system (GIS) methodologies has been used effectively as a rational approach with which to model and predict the risks associated with the various mining-induced hazards. The purpose of this study was to review the GIS-based methods and applications currently used for the modeling and assessment of mining-induced hazards associated with soil, water, and forest. Other mine hazards, such as subsidence, slope failure, gases, and abandoned facilities, were excluded in the review in this study.

GIS is a computer-based technology and methodology for collecting, managing, analyzing, modeling, and presenting geospatial data for a wide range of applications [3]. Recently, geospatial data related to various events have become increasingly diverse and complicated. Thus, GIS has often been combined with analytical models and methods (e.g., probability/statistical, machine learning and data mining methods) to complement the inherent capabilities of GIS in evaluating the spatial patterns or characteristics of the events and their attributes.

The scope of this review paper was confined to published literature concerning GIS-based modeling and assessment of mining-induced soil, water, and forest related hazards that included all the following keywords (or concepts): GIS, mine, type of hazard. For soil-related problems within mining areas, the keywords “soil contamination” or “soil erosion” were used to search and review the literature. Articles concerning soil in urban areas and commercial districts, and soil problems caused by shooting ranges or other activities, were not taken into account. Many researchers have collected samples from soil, stream sediment, and/or water and they have presented elemental concentration data for specific points on a map. However, such studies lack a GIS analytical element and thus, they were excluded from this study. For problems concerning water in mining areas, including tailing dam sites, the terms “water pollution” or “drainage” were used as keywords. The terms “deforestation” or “reforestation” were used in relation to hazards associated with forests in mining areas. Numerous studies have reported on the detection and monitoring of acid mine drainage (AMD), water pollution, and changes in forest area in mining areas using remote sensing observational technologies (e.g., hyperspectral sensors and InSAR) or unmanned aerial vehicle (UAV) photogrammetry. However, these phenomena were considered beyond the scope of GIS technology and they were excluded from this study.

## 2. Mining-Induced Hazards of Soil, Water, and Forest

The lifecycle of a mine typically includes four phases: exploration and feasibility, planning and construction, operation, and closure [4]. In the exploration and feasibility phase, economic deposits are identified and their characteristics are determined to allow recovery. In planning and construction, preparations are made for mine development. In the operation phase, valuable material is extracted for processing or sale. In the closure phase, activity ceases and the area is abandoned or returned to another use [5]. Each phase of the mining life cycle includes various activities, all of which have potential environmental concerns or hazards (Table 1).

Potential toxic elements (PTEs) in the soil at mine sites pose a risk to human health because of their potential to enter the food chain via direct ingestion of dust or the ingestion of plants [6]. Consequently, their presence in high concentrations in the soil at mine sites is clearly a matter of concern. Knowing the extent and the level of contamination of the soil by identifying the spatial distribution of PTEs from soil contamination maps is a crucial step toward the management and alleviation of soil contamination with elevated levels of PTEs.

Water management and drainage control at a mine site represent important issues regarding control operational processes, hazard prevention, and secondary hazard prevention. Open-pit mines are affected directly by rainfall and, in particular, surface water control is important for such mines in humid regions [7]. Moreover, underground mines could be submerged because of rainfall, which is recognized as a major problem in terms of mine operation and safety. To prevent water-related mining hazards, it is necessary to control water pollution in relation to operational and abandoned mines [8].

Changes in forest cover have attracted worldwide attention because of their potential effects on soil erosion, runoff, and atmospheric carbon dioxide levels [9]. Mining is an obvious cause of deforestation. It can destroy landscapes, forests, and wildlife habitats, which can lead to soil erosion. Large-scale mining operations, especially those using open-pit mining techniques, can result in significant deforestation through forest clearance and the construction of mining facilities and roads. Environmental degradation and deterioration due to mining activities are major problems in many countries throughout the world [10]. Many countries require mining companies to implement reclamation plans to repair the damage to their forests; therefore, it is important to understand deforestation within the spatial and temporal domains, and to support reclamation plans within a geospatial perspective.

As listed in Table 1, in a broad sense, the closure phase of the life cycle of a mine includes the reclamation works that correct or ameliorate disturbances caused by any of the preceding activities. However, if appropriate reclamation measures are not implemented during the closure phase, various mine-related hazards that occurred during the preceding phases could remain present at an abandoned site, even after closure. These mining hazards could have direct, distinct, and long-lasting effects both on the physical environment and on human health.

## 3. Soil Contamination and Erosion in Mining Areas

The subject of soil problems in mining areas is generally classified into pollutant transport based on hydrological analysis, geostatistical spatial interpolation for soil contamination mapping, and sediment yield from mine tailing dumps.

### 3.1. Pollutant Transport Modeling Based on Hydrological Analysis

Pollutant transport in soil is strongly associated with local hydrological characteristics. Thus, the spatial variations of heavy metal enrichments in soils might be related to natural dispersion processes such as leaching by percolating rainwater or mechanical transport in runoff.

Hwang and Kim [11] examined the distribution patterns of trace elements in stream sediments near coal mining areas using factor analysis and GIS. To compile drainage-based geochemical maps, catchment basins were calculated from digital elevation models (DEMs) and streamline segments. The streamline segments and sampling points were also considered as a target image for watershed segments, and it was found that streamline segments were better at reflecting the catchment area than the sampling points.

Yenilmez et al. [12] determined pollution levels at an abandoned coal mine site with the aid of GIS tools by evaluating the spatial distributions of pollutant concentrations with respect to surface runoff pathways and the locations of potential contamination sources such as open pits, and coal storage and dump sites. It was observed that pollutant concentrations were higher closer to the contamination sources and along the surface runoff pathways. Results indicated that GIS could help locate areas most likely to have high concentrations of pollutants. This could help prevent overlooking highly contaminated points located far from contamination sources. Moreover, these areas could be determined using a smaller number of samples (near the surface runoff pathways), which would decrease sampling costs. It is unnecessary to investigate all the area far from contamination sources.

**Figure 2:**
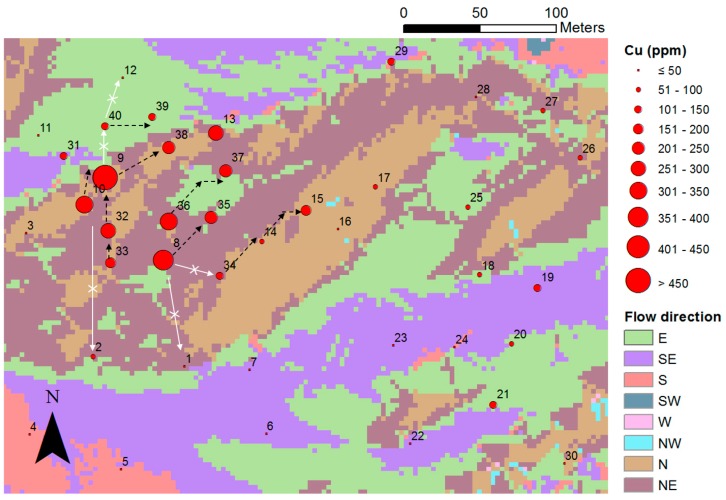
DEM-based hydrological analysis used to examine the effect of the single-flow direction of rainwater on Cu dispersion (modified from Suh et al. [13]).

Suh et al. [13] performed DEM-based hydrological analysis for the evaluation of the effect of single-flow direction of surface runoff on Cu dispersion. This study analyzed the single-flow direction of rainwater over the entire study area, based on local topographic relief, and it compared it with the distribution of Cu concentration at sampling points (Figure 2). The results revealed that the dispersion pattern of soil contaminants was influenced by the single-flow direction of rainwater, even though the distinct high-level contaminants (pollution sources) could not be determined within the study area. This finding could assist in selecting additional sampling points for further investigation or validation.

### 3.2. Soil Contamination Mapping Using Geostatistical Spatial Interpolation

Geostatistical spatial interpolation and simulation methods can be utilized to generate raster grid-cell-based soil contamination maps and to explore the spatial variations of heavy metal pollution. Soil contamination maps enable the natural background levels for an area to be distinguished from the anomalous anthropogenically enriched levels, and to identify areas of contaminated topsoil that require remedial action.

Nakayama et al. [14] quantified the concentrations of six metals and of one metalloid in roadside soils and wild rats found around a Pb–Zn mine (Kabwe, Zambia) and Lusaka, the capital city of Zambia, and they analyzed the source of metal pollution using GIS. The concentrations of Pb, Zn, Cu, Cd, and As in the Kabwe soil were much higher than benchmark values. GIS-based analysis and mapping indicated the source of metal pollution was mining and smelting activity. Wild rats from Kabwe had much higher tissue concentrations of Pb than those from Lusaka. Their body weights and renal Pb levels were negatively correlated, which suggests that mining activities might have affected terrestrial animals in Kabwe.

Dong et al. [15] modeled heavy metal distribution using geostatistical analyses, and they determined the ecological safety of land reclaimed for agricultural purposes from subsided areas that had been filled with mining waste and fly ash. Among the six elements studied (i.e., As, Hg, Pb, Cu, Cd, and Cr), seriously high levels of Cd were found in the reclaimed soils and in control soil at different depths. A Kriging interpolation model was applied to investigate the regular distribution of Cd at each site. Subsequently, using a polynomial model of measured concentration data at different depths, the Cd concentrations were calculated at four different depths.

Khalil et al. [16] assessed soil contamination around an abandoned mine in a semiarid environment using geochemistry and they elaborated geochemical maps using a simple Kriging (SK) method within a GIS environment. The geochemical background was determined based on exploratory data analysis for five elements of interest. The obtained results showed that Kettara soils are contaminated with metals and a metalloid at levels that exceed the established geochemical background values (Cu = 43.8 mg/kg, Pb = 21.8 mg/kg, Zn = 102.6 mg/kg, As = 13.9 mg/kg, and Fe = 56,978 mg/kg). Geochemical maps showed that deposited mine wastes were responsible for the soil contamination, because the released metals and metalloid were dispersed downstream from the mine waste dump via water transport after rainfall.

Reis et al. [17] combined GIS and stochastic simulation to estimate the spatial distribution of Pb and to assess the quality of soil at a mine in Portugal. A p-field simulation produced numerous sets of equiprobable realizations for Pb concentrations, meaning there were numerous equiprobable scenarios responsible for the spatial distribution of the pollutant element. These equiprobable realizations indicated that the simulated values did not exceed the risk-based standards used to assess soil quality. The resulting probability maps were coded into binary maps for the purposes of delineating areas hazardous to human health and classifying soil quality. Finally, analysis of the influence of topography on the dispersion of the metal, made possible using GIS techniques, has allowed better perception of the mechanisms controlling the spatial distribution of the metal. The results obtained showed some advantages of stochastic simulation over ordinary Kriging (OK).

Acosta et al. [18] evaluated the behavior of heavy metals at mine sites with regard to future land reclamation using a multivariate statistical and GIS-based approach. A GIS-based approach was adopted to examine the spatial distributions of waste properties and heavy metals, and to identify sites of highest risk where most reclamation and monitoring efforts should be realized. As a result, according to their environmental risk, five locations at northern, southern and western edges of the tailing pond were selected.

Yan et al. [19] estimated the spatial distribution pattern of heavy metals and assessed moso bamboo forest soil near lead-zinc mine in Southeastern China using multivariate statistics and Kriging interpolation method. The result showed that most of the soil heavy metals represented a geographically increasing concentric trend and Pb concentration in bamboo shoots exceeded the standard limits. In addition, approximately 60% of the study area suffered from metal contamination.

Lee et al. [20] compared the prediction performances of four different approaches for two different geostatistical mapping techniques of Cu and Pb concentrations at abandoned mining areas using element analysis data from Inductively Coupled Plasma–Atomic Emission Spectroscopy (ICP–AES) and Portable X-ray fluorescence (PXRF) instruments: (1) OK to ICP–AES analysis data; (2) OK to PXRF analysis data; (3) OK to both ICP–AES and transformed PXRF analysis data by considering the correlation between the ICP–AES and PXRF analysis data, and (4) Co-Kriging (CK) to both the ICP–AES (primary variable) and PXRF analysis data (secondary variable). When compared against an independent validation data set, the results showed that the applications of OK to both ICP–AES and transformed PXRF analysis data were the most accurate approach when considering the spatial distributions of Cu and Pb contaminants in the soil and the estimation errors at 11 sampling points for validation. From the results, this study revealed that it is beneficial to use the proposed approach that incorporates the advantageous aspects of both ICP–AES and PXRF analysis data when generating soil contamination maps for an abandoned mine.

**Figure 3:**
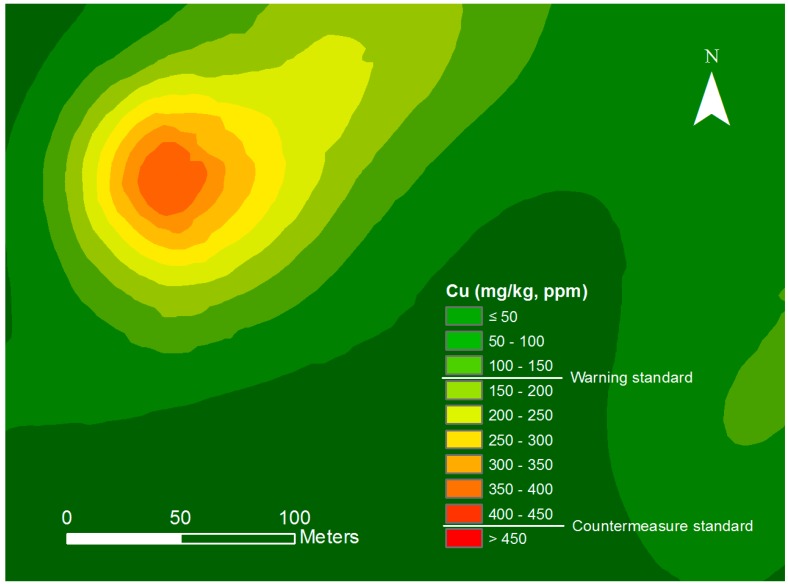
Spatial distribution map of Cu with levels of soil contamination exceeding the Korea Soil Contamination Warning Standards or Countermeasure Standards (modified from Suh et al. [13]).

Suh et al. [13] proposed a rapid, accurate, and efficient method to investigate and map soils at mine sites contaminated by heavy metals using converted PXRF data and geospatial interpolation within a GIS environment. This study analyzed the prediction accuracy and time required for mapping Cu concentrations in soil. Consequently, the suggested method significantly shortened the time required for mapping compared with conventional mapping methods (by 95%, i.e., only approximately 8 h needed for collecting and analyzing 40 samples), and it provided Cu concentration estimates with high accuracy similar to those measured by ICP–AES (R^2^ = 0.9997). In addition, this study delineated the extent and calculated the areas with levels exceeding the warning standards and countermeasure standards of Cu from the raster grid-cell-based soil contamination map (Figure 3).

Kim et al. [21] developed a new Kriging method to predict heavy metal concentrations in stream sediments. Their proposed method compensated the drawbacks of Kriging based on Euclidean distances because it used the stream distance for the prediction by analyzing the stream path and networks using a DEM. Moreover, the developed method could reduce the overestimation problem in predicting the concentration of an uncontaminated stream segment by considering the catchment basin area in the Kriging.

### 3.3. Soil Erosion and Sediment Yield

GIS can be combined with the Universal Soil Loss Equation (USLE) model [22] to estimate soil erosion from a mine tailing dump for a specific mine region. Kim et al. [23] estimated soil erosion and sediment yield from the mine tailing dumps of abandoned mining areas using GIS and the USLE model. Using a GIS database and the mean annual rainfall over 30 years (recorded at the nearest observatory), this study processed and compiled maps of the five major factors that affect soil erosion: the rainfall erosivity factor (R, in MJ mm ha^−1^ h^−1^ year^−1^), soil erodibility factor (K, in ton h 107 J^−1^ mm^−1^), slope length and steepness factor (LS), cropping management factor (C), and supporting conservation practices factor (P). Subsequently, by multiplying the maps of these major factors using a map algebra technique, the mean annual rate of soil erosion (A, in ton ha^−1^ year^−1^) was calculated as follows:

A = R × K × LS × C × P(1)

**Figure 4:**
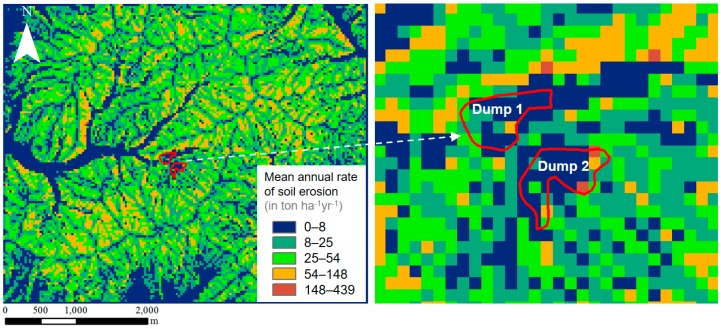
Mean annual rate of sediment yield (in ton ha^−1^ year^−1^) from each mine tailing dump using GIS-based spatial analysis and the USLE model [23].

The soil erosion and sediment yield from mine tailing dumps within the study area were calculated as 75.63–350.24 tons year^−1^ and 40.40–187.64 tons year^−1^, respectively (Figure 4). Kim et al. [2] developed a new GIS extension, named the ArcMine mine waste erosion tool, to perform rapid estimations of the erosion of mine waste dumps in abandoned mining areas. This software calculates the USLE factors and it estimates soil erosion over the entire region of interest. A DEM, land cover map, soil map, and annual rainfall data are all used as inputs. The R factor is calculated from the annual rainfall data, the K factor is determined according to the soil series, the LS factor is computed from slope length and slope derived from the DEM, the C factor is calculated from the land cover map, and the P factor is derived according to the slope and the cultivation condition. By multiplying the five input raster maps over the entire grid, the soil and mine waste erosion value can be estimated.

## 4. Water Pollution and Drainage Control

The subject of water problems in mining areas was classified into AMD runoff analysis, flooding modeling, and spatial analysis for drainage control.

### 4.1. Water Pollution in Mining Areas

The AMD caused by flooding of a mine translates along the terrain and it results in water pollution to the surroundings. Studies have been performed to support mine reclamation by estimating AMD pathways.

**Figure 5:**
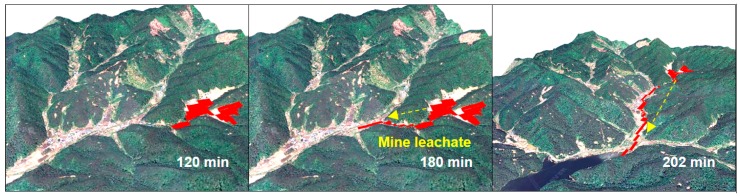
DEM grid-based hydrological mine leachate transport modeling with time: 120, 180, and 202 min [24].

Yenilmez et al. [12] estimated the route of AMD using the single-flow direction method, flow accumulation, and catchment area analysis. This study compared the results with water and soil samplings and it discussed the distributions of the contaminants. Kim et al. [2,24,25] proposed a technique to model the temporal pathway of AMD (Figure 5). The underground absorption of surface runoff was applied to effective rainfall theory. In addition, the results of GIS-based algorithms for calculating the single-flow direction in a DEM were examined by concave- and convex-shaped topography, and software was developed to allow users to apply the algorithms effectively. Yi et al. [26] suggested a technique to propose AMD monitoring points, by identifying where the water systems meet, with consideration of the shape of the stream. Furthermore, a method to evaluate putative AMD sources based on mine location and monitoring points was designed in the study. Norman et al. [27] investigated the traditional model used to estimate erosion and sediment deposition to assess the potential risk of water quality impairment related to mining works. GIS-based watershed analysis was performed to identify erosion and sediment transport characteristics within the catchment areas. The result showed that the AMD occurred in the study area due to the proximity of mines to the surface-flow discharge. It was found that sediment in streambeds contributed from mined areas is linked to poor water quality.

### 4.2. Inundation in Mining Areas

Inundation in a mine occurs through gaps in the drift in underground mines, and in open-pit mines according to terrain, runoff, and the behavior of the water treatment facility. Studies have been conducted to simulate these phenomena and to evaluate the associated risk.

**Figure 6:**
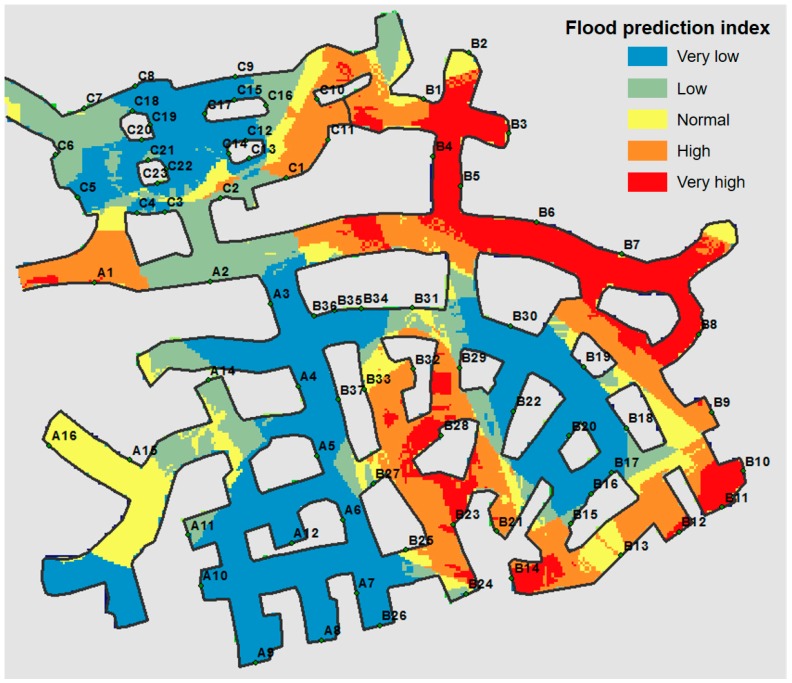
Flood prediction map of underground limestone mining area with five categories using the FR model based statistical analysis of the five data elements (modified from Park et al. [28]).

For example, Park et al. [28] proposed a method for determining a zone of caution regarding flooding in an underground mine using a Frequency Ratio (FR) model within a GIS environment. They used five data elements as indicators: rock mass rating, Q value, distance from faults, depth of mine drift, and flow accumulation. The study provided a local-scale flooding area (Figure 6), which could be used as a basis for the stability assessment of an underground mine.

Yi et al. [29] developed an algorithm for modeling the temporal inundation area in an open-pit mine site. This study used a DEM and infrastructure for simulating inundation phenomena, effective porosity, saturated hydraulic conductivity, pore size distribution index, and degree of initial absorption by applying Philip’s two-term infiltration model and precipitation for scenario analysis.

Kong et al. [30] obtained numerous investigation data on the flood risk about 3 months in the Wuda Coal Mine, Inner Mongolia using GIS and remote sensing, and global positioning system in combination with some field work.

### 4.3. Drainage Control in Mining Areas

GIS analysis based on surface water movements in mining areas can also be used to design the placement, capacity, and networks of drainage control infrastructure such as pumps, safety berms, and pipes. Various studies have been conducted for this purpose [31,32,33,34,35].

Choi et al. [31] used approximate expressions for rising water level and pump capacity to propose a formula for water level maintenance. The proposed formula could be applied to the drainage design of an open-pit mine, but it does not reflect that the area of pond could change according to rainfall. Sunwoo et al. [32] modeled the formation of a water system for open-pit mine, and they proposed a location where a drainage line or safety berm should be installed. For the modeling of the water system formation, the single-flow direction method, flow accumulation, and catchment area analysis were applied. Song et al. [33] designed the allocation of pumping facilities for a tailing dam. This study analyzed the risk of flooding according to the operation of some or of all the pumping facilities through scenario analysis. To improve the reliability of the results, an effective rainfall model was applied to the rainfall data. Choi et al. [34] proposed the placement of a pump facility and estimated the flooding risk according to the facility capacity for a coal mine. Choi and Park [35] improved the GIS technique for the drainage design of open-pit mines. To reflect the change of terrain according to the rise of water level, a technique of modifying the DEM was proposed.

**Figure 7:**
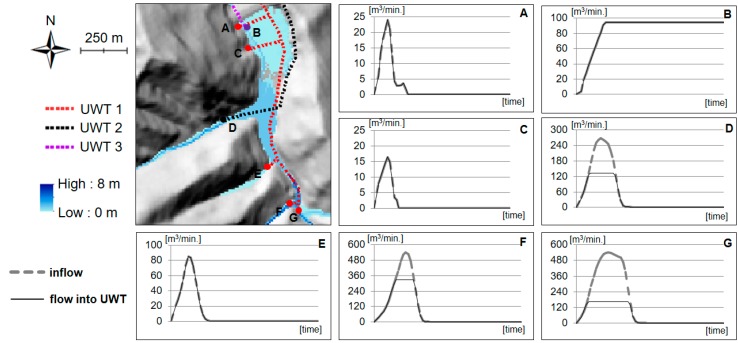
Time-specific inflow and flow into storm sewer collection systems (**A**–**G**) considering ground and underground water pipelines (modified from Yi et al. [29]). UWT refers to underground waterway tunnel. Elevation indicates water level. Inflow refers to the water volume to the grid cell. Flow into UWT indicates water volume to the UWT, which may smaller to Inflow due to the capacities of the UWT.

Choi et al. [36,37] developed an adaptive stormwater infrastructure (ASI) algorithm that considers ground and underground water pipelines in the process of GIS-based hydraulic analysis. This algorithm was released and applied to scenarios according to the operation of facilities in a tailing dam area [38]. However, these studies did not suggest an accumulative runoff over time for the inlet and outlet of the water treatment facility. Yi et al. [29] improved the ASI algorithm presented previously. This study developed an algorithm to simulate both the temporal inflow and the amount of flux reaching the inlet of the underground waterway tunnel (Figure 7). This algorithm could be utilized in facility capacity design in mining areas.

## 5. Deforestation in Mining Areas

GIS-based research of forest cover at mining sites was classified into three topics according to the steps of reclamation: deforestation, decision support for reforestation, and reforestation.

### 5.1. Assessment of Deforestation in Mining Areas

A GIS technique involving remote sensing data can be used as a tool for assessing and monitoring deforestation resulting from mining activities. GIS has a distinct advantage in mapping and monitoring the evolution of degraded areas.

Prakash and Gupta [39] used remote sensing and GIS techniques for the identification of various land use classes based on satellite imagery and sequential changes of land use patterns in the Jharia coalfield (India). Normalized difference vegetation index (NDVI) images, which show the density of plant growth over an area, have been used widely for vegetation studies. Salyer [40] analyzed the change in vegetation across Wise County (VA, USA), which is an area that encompasses 12 active mining sites. Coal production in this area is achieved through auger, deep, and surface mining methods. Landsat satellite images were analyzed to derive an NDVI. To confirm that mining is in fact the cause of the vegetation loss, the overall change in the state of the vegetation of the study area was calculated using Erdas Imagine’s Change Detection function and ArcMap’s Spatial Analyst extension for specific mining methods. Mag-usara and Japitana [41] also identified land cover changes due to mining using an NDVI based on Landsat satellite images of Carrascal, Surigao del Sur (The Phillipines) by employing object-based image analysis. To minimize the classification error and to maximize the delineating margin, the support vector machine approach was used to optimize the separating parameters for each class. The results showed that forest cover had decreased by 14.46% and that a significant amount of forest had been converted into barren land.

### 5.2. Decision Support for Reforestation in Mining Areas

GIS technology can be utilized to support a decision for reforestation after a deforestation assessment. Post-mining land restoration is one of the important aspects of an environmental management program, and spatial data inputs can be analyzed using GIS for the planning and design of reforestation programs.

Perera et al. [42] produced a map of southern Sri Lanka, which shows areas most suitable for reforestation after analyzing Landsat satellite data, river and road networks, open–water areas, and rainfall and temperature information using GIS technology. A number of buffer zones, selected as key areas, were merged with other GIS files through a specially arranged point system. A management information system was developed as part of the ASTERISMOS project that supports mining companies in evolving restoration plans tailored to specific situations [43,44]. Such a system can assist mining experts to develop various reforestation alternatives and to explore both the environmental and the economic effects of reforestation alternatives. The system uses multicriteria analysis (MCA) to allow the user to set weights for different objectives and to calculate the optimal solution. Remote sensing is used to obtain all relevant data on environmental conditions such as land cover and land cover change, together with other geographical data such as geology and soil type, which are all stored in an accessible GIS database.

**Figure 8:**
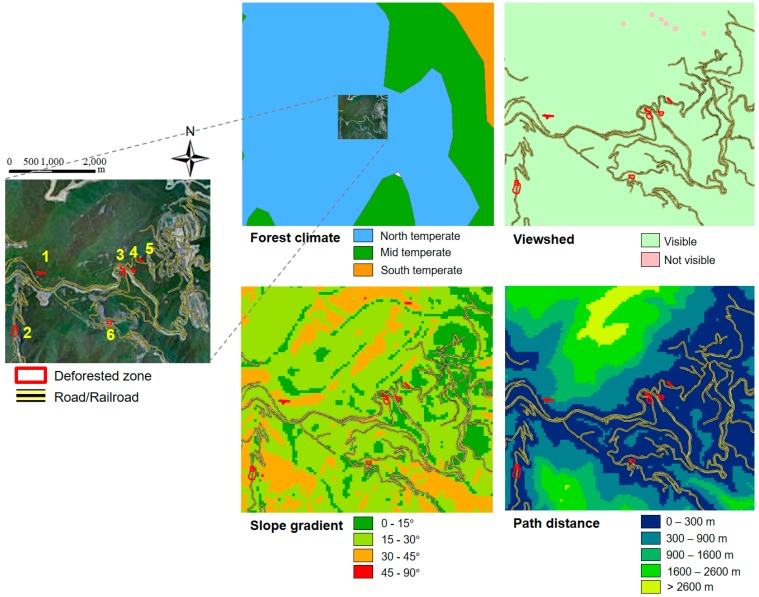
GIS-based reforestation planning for deforested areas using forest climate map, viewshed analysis map, slope analysis map, and path distance map (modified from Kim et al. [2]).

Choi et al. [45] classified deforested areas by considering various conditions such as topography, geology, and climate to identify tree species suitable for reforestation at abandoned mining sites. This study used GIS-based spatial analysis to consider criteria such as forest climate type, visibility, slope gradient, managerial condition, mining method, and reforestation purpose to classify the types of deforested areas. Oh et al. [46] developed a system that estimates the costs for reforestation based on these criteria using ArcMap, ArcObjects, and Visual Basic.NET. This system was integrated with other GIS-based mine hazard analysis tools into a single framework, ArcMine [2]. Figure 8 shows the process of reforestation planning by analyzing various conditions based on GIS data of deforested areas. Galan et al. [47] constructed a reforestation model using Bayesian networks for pattern recognition. The model was trained using data of existing wooded areas to serve as a guideline for the reforestation of deforested areas. The model determines the importance of the variables for reforestation such as altitude, slope, potential insolation, lithology, precipitation, and distance from the sea.

Kisan et al. [48] generated maps of soil erosion and surface runoff potential for a region associated with iron ore mining within the Saranda Forest in Jharkhand (India) to identify and prioritize locations for reforestation. The Analytic hierarchy process (AHP) technique was used for recommending specific sites for reforestation strategies with the help of the USLE and the Soil Conservation Services Curve Number (SCSCN) Method using remote sensing and GIS tools. Trabucchi et al. [49] presented an approach for incorporating the assessment of ecosystem services and a key ecological degradation factor for prioritization of reforestation sites. This study analyzed the spatial distribution of critical services such as water flow regulation, carbon storage in woody vegetation, erosion prevention, maintenance of soil fertility, and potential recreation and ecotourism of a river basin in Northeastern Spain, where widespread coal mining had caused deforestation and the full removal of topsoil.

### 5.3. Assessment of Reforestation in Mining Areas

GIS techniques using remotely sensed data can be applied to assess the results of reforestation. Joshi et al. [9] used remote sensing and GIS-based techniques for deforestation and reforestation estimation in the Korba coalfield (India) by applying data preprocessing, interpretation, and change analysis. Landsat Multispectral Scanner, Thematic Mapper, Enhanced Thematic Mapper, and ResourceSat-1 Linear Imaging Self Scanning Sensor III digital data were used to evaluate the changes. Temporal NDVI images were used to detect the mining area and to trace the areas already reclaimed.

Malaviya et al. [50] also assessed the impact of coal mining and reclamation on the forest cover of the Bokaro District of Jharkhand (India) using geospatial tools in conjunction with landscape metrics. It was confirmed that just a small part (0.26%) of the study area has been reclaimed successfully. This study demonstrated the potential of using remotely sensed data and GIS, integrated with landscape parameters, in monitoring post-mining landscapes and reforestation or reclamation activities. It assessed the effectiveness of reforestation by capturing erosion scars on aerial photography taken before and after reforestation, and any change in scar size was measured within the GIS environment. This study also modeled sediment yield, and it demonstrated that declining sediment production from reforested areas would likely reduce the incidence both of damage to structural utilities and of flooding of the floodplain.

## 6. Advantages and Limitations of GIS in Mine Hazard Studies

To handle mining-induced geohazard related data, GIS is appropriate for the following reasons:

- Concurrent handling of spatial and attribute data: Mining-induced hazard assessment has to deal with information comprising spatial data (locational characteristics of objects) and attribute data (property-related characteristics of objects). GIS data can be represented in separate layers.
- Variety of geospatial data: Mining-induced hazard assessment often requires various forms and types of data (e.g., topographic contour maps, mine drift maps, geological maps, hydrological maps, hazard inventory maps, factor-derived maps, tables of diverse observations, and data sets). Mostly, the sources of these data could be aerial photographs or satellite images, field surveys, and laboratory analyses, and they are usually in the forms of digital maps, tables, and figures.
- Flexibility of operations and concurrent display: GIS packages or software offer numerous data processing functions, data management tools, and data analysis capabilities in a highly flexible manner with a concurrent interactive display facility.
- Fast and inexpensive processing: GIS can efficiently store, process, analyze, and visualize large volumes of spatial data, which otherwise would be too expensive, tedious, and time-consuming to conduct by other methods.
- High accuracy and repeatability of results: The technique is based on digital mapping and it yields higher accuracy compared with manual cartographic products. In addition, the records and results are suitable for rechecking and confirmation.

Because GIS can also be considered computer-coded mapology, it can easily represent and combine factor maps and therefore, effectively derive susceptibility, hazard, and risk indices as well as perform modeling efficiently. Therefore, GIS is considered very important in the entire process of mining hazard modeling and mapping production. In addition, GIS-based geohazard mapping based on other concerned geospatial data sets can provide basic data both for engineers and for planners to assist them in making decisions.

Although GIS is a useful tool, GIS-based approaches remain challenging to model and assess the mining-induced hazards. The inaccuracies and imprecisions of input data can affect the quality of the resultant map. Most of previous GIS-based studies employed 10–30 m spatial resolution to analyze and mapping mining-induced hazards because it is appropriated for the local or regional scale analysis and mapping (not for site specific). However, aforementioned spatial resolution of the data may have inaccuracies (attribute values differ from the original properties) and is too coarse to precisely model and map the flow, spread, estimation of the actual mining hazards. Therefore, it is necessary to use more high-resolution input data to obtain better results.

## 7. Conclusions

In this paper, various types of mining-induced hazard and the strength and role of GIS as a geospatial problem-solving tool were introduced. Furthermore, numerous examples of published literature on GIS-based modeling and assessment of several representative mining-induced hazards were discussed. To focus on papers dealing with GIS analysis techniques for the modeling and assessment of mining hazards, remote sensing technology based research was excluded in this study. For the detailed review of the numerous papers, each area of mining hazard research (e.g., soil contamination, water pollution, and deforestation,) was classified into two or three subtopics according to the steps of reclamation procedure, or element of hazard of interest (Table 2).

(1) Soil contamination maps provide information regarding hazardous surface soils by representing the distribution of elevated levels of contaminants within the soils. This can assist both in prioritizing the abandoned mines that pose the greatest risk, and in deciding on appropriate remediation measures to prevent the spread of soil contaminants (in particular, areas exceeding contamination warning standards or countermeasure standards for the different PTEs). In this sense, these types of studies are helpful both to engineers and to planners involved in the design and implementation of efficient soil management strategies in abandoned mining areas.

To obtain inexpensive and accurate soil contamination maps, efforts to increase the number of sampling sites and to improve the prediction accuracy of interpolation techniques are needed. The ICP-AES method is accurate but expensive, whereas the PXRF method is rapid (low cost) but relatively inaccurate in the measurement of PTEs in soil. Numerous studies have shown high correlation between ICP-AES and PXRF analysis data for most elements present in soil [13,20]. Thus, if sufficient in situ PXRF data were available, together with a database of correlation, it would be possible to obtain a large quantity of sampling analysis data and a reliable soil contamination map. Therefore, the construction of a database on the correlation between ICP-AES and PXRF analysis data for PTEs is necessary.

It is evident that the prediction accuracy of a soil contamination map could be improved by increasing the number of sampling sites; however, attempts to increase prediction accuracy by improved geospatial interpolation techniques using confined samples should also be considered. The spatial variability of PTEs could be changed easily because they tend to be dispersed or leached by rainfall or by the rise of groundwater level. In particular, several GIS-based pollutant transporting modeling studies have revealed that the dispersion pattern of contaminants is highly affected by the single-flow direction of surface rainwater. Nonetheless, many previous soil contamination studies have not considered the effect of the single-flow direction of surface rainwater in the interpolation process of generating a soil contamination map. This is because of the inhomogeneity of the hydrological tendency and the difficulty of synthetic interpretation. In future work, it will be necessary to consider the hydrological characteristics as part of the geostatistical interpolation process to analyze the spatial pattern of contaminants for better prediction of the distributions of PTEs in the soil at mine sites.

(2) GIS-based studies of water pollution in mining areas have progressed gradually. These studies have increasingly taken into account a greater number of variables and they have derived time-series analysis results. In addition, improved simulation accuracies of the fluxes by rainfall or streams have been developed, and the simulations of the resulting phenomena have become more precise. The types of algorithm applied have also diversified and the hazards have been simulated more realistically, providing improved results for drainage design. Nevertheless, further studies are needed. A method for quantitative flood assessment is required for underground mining sites in flooding modeling. There is also an area for improvement in the modeling of the behavior of ground drainage sewers in surface areas. This could be achieved by algorithm improvement and by proposing techniques for using ultrahigh-resolution DEMs derived from UAVs. Moreover, if collaboration with the field of groundwater modeling were achieved, it would improve the temporal simulation of runoff by linking surface and underground water movements.

(3) GIS technology can be widely applied to all of the forest reclamation procedures in mining areas such as deforestation assessment, decision support systems for reforestation, and reforestation assessment and design. Although GIS-based decision support systems for forest restoration in mining areas have been proposed, the economic and environmental improvements realized by applying these methods to actual mining sites are insufficient. Changes in the land cover of forest areas in mining sites are associated with other hazards such as landslides, and soil erosion. Therefore, it is necessary to evaluate the use and effectiveness of GIS technology by analyzing the overall process including the evaluation of the degree of forest degradation, application of forest recovery methods, and assessment of the outcome of forest restoration.

The widespread use of GIS has significantly improved the modeling and assessment capabilities of mining-induced hazards associated with soil, water, and forest. Furthermore, related research continues to improve and develop via the use of ultrahigh-resolution geospatial data, advancement of spatial data analysis techniques, and coupling of GIS technology with various (empirical, theoretical, and analytical) models and methods. However, a gap is present between the derivation of maps (by researchers) and their practical use in mine reclamation and management works (by engineers, planners, and designers). Consequently, greater efforts are required to minimize this gap by maximizing the applicability and practicality of future modeling and assessment results.

## Figures and Tables

**Table 1:** Mining-induced hazards according to the mine life cycle (modified from Environment Canada [4]).

Phase	Key Activities	Mine Hazard
Exploration & Feasibility	· Reconnaissance; locate mineral anomalies · Discovery, sampling · Decision about economic feasibility of mining	· Deforestation · Noise · Vibration
Planning & Construction	· Mine planning · Environmental/social planning · Closure plan · Environmental assessment · Environmental and other permits · Clearing, stripping, blasting; infrastructure	· Waste rock · Deforestation · Noise · Vibration
Operations	· Ore extraction · Crushing, grinding, concentrating · Waste rock and tailings management · Wastewater management · Progressive reclamation	· Subsidence · Soil contamination · Waste rock, tailing · Water pollution (AMD) · Slope failure · Noise · Vibration
Closure	· Site cleanup; reclamation; rehabilitation · Maintenance; environmental monitoring	· Subsidence · Soil contamination · Water pollution (AMD) · Deforestation · Slope failure

**Table 2:** Classification and summary of research concerning GIS-based modeling and assessment of mining-induced hazards associated with soil, water and forest.

Classification	Sub-Classification	Technique	Country	Reference
Soil contamination & erosion	Pollutant transport modeling based on hydrological analysis	Watershed analysis	Korea	Hwang and Kim [11]
		Surface runoff analysis	Turkey	Yenilmez et al. [12]
		Single flow direction	Korea	Suh et al. [13]
	Soil contamination mapping using geostatistical interpolation	OK	Korea	Suh et al. [13]
		OK	Zambia	Nakayama et al. [14]
		OK	China	Dong et al. [15]
		SK	Morocco	Khalil et al. [16]
		Indicator Kriging, Stochastic simulation	Portugal	Reis et al. [17]
		Inverse distance weighting	Spain	Acosta et al. [18]
		OK	China	Yan et al. [19]
		OK & CK	Korea	Lee et al. [20]
		Catchment Kriging	Korea	Kim et al. [21]
	Sediment yield from mine tailing dumps	USLE model	Korea	Kim et al. [2]
		USLE model	Korea	Kim et al. [22]
Water pollution	AMD runoff analysis	Single flow direction	Turkey	Yenilmez et al. [12]
		Single flow direction & effective rainfall theory	Korea	Kim et al. [2,24,25]
		Single flow direction & temporal flow accumulation	Korea	Yi et al. [26]
		Watershed analysis	USA	Norman et al. [27]
	Flooding modeling	FR statistical model	Korea	Park et al. [28]
		Time-specific accumulative flux of surface runoff	Korea	Yi et al. [29]
		Flooding simulation	China	Kong et al. [30]
	Drainage control using spatial analysis	ASI algorithm	Korea	Yi et al. [29]
		Single flow direction & temporal flow accumulation	Indonesia	Choi et al. [31]
		Single flow direction & catchment area	Indonesia	Sunwoo et al. [32]
		Effective rainfall model	Korea	Song et al. [33]
		Temporal flow accumulation & catchment area	Indonesia	Choi et al. [34]
		Temporal flow accumulation & catchment area	Indonesia	Choi and Park [35]
		ASI algorithm	Korea	Choi et al. [36,37]
		Weighted ASI algorithm	Korea	Choi [38]
Deforestation	Deforestation	NDVI change detection	India	Prakash and Gupta [39]
		NDVI change detection	USA	Salyer [40]
		NDVI change detection	Philippines	Mag-usara and Japitana [41]
	Decision support system for reforestation	Land suitability analysis	Sri Lanka	Perera et al. [42]
		MCA	Spain	De Vente and Aerts [43]
		MCA	Greece	Ganas et al. [44]
		MCA-based decision support system	Korea	Choi et al. [45]
		MCA-based decision support system	Korea	Oh et al. [46]
		Bayesian networks for pattern recognition	Spain	Galan et al. [47]
		AHP, USLE model, SCSCN	India	Kisan et al. [48]
		MCA	Spain	Trabucchi et al. [49]
	Reforestation	NDVI change detection	India	Joshi et al. [9]
		Change detection analysis of scar size	India	Malaviya et al. [50]

## Abbreviations

The following abbreviations are used in this manuscript:

GIS	Geographic information systems
AMD	Acid mine drainage
UAV	Unmanned aerial vehicle
PTEs	Potential toxic elements
DEM	Digital elevation model
SK	Simple Kriging
OK	Ordinary Kriging
CK	Co-Kriging
ICP–AES	Inductively Coupled Plasma–Atomic Emission Spectroscopy
PXRF	Portable X-ray fluorescence
USLE	Universal Soil Loss Equation
FR	Frequency Ratio
ASI	Adaptive stormwater infrastructure
NDVI	Normalized difference vegetation index
MCA	Multicriteria analysis
AHP	Analytic hierarchy process
